# The Management of Psychomotor Agitation Associated with Schizophrenia or Bipolar Disorder: A Brief Review

**DOI:** 10.3390/ijerph18084368

**Published:** 2021-04-20

**Authors:** Maurizio Pompili, Giuseppe Ducci, Alessandro Galluzzo, Gianluca Rosso, Claudia Palumbo, Domenico De Berardis

**Affiliations:** 1 Department of Neuroscience, Mental Health and Sensory Organs, Suicide Prevention Center, Sant’Andrea Hospital, Sapienza University of Rome, 00189 Rome, Italy; 2 Mental Health Department, ASL Roma 1, 00193 Rome, Italy; giuseppe.ducci@aslroma1.it; 3 Department of Mental Health and Addiction Services, ASST Spedali Civili, 25123 Brescia, Italy; alessandro.galluzzo@asst-spedalicivili.it; 4 Psychiatric Unit, San Luigi Gonzaga University Hospital, 10043 Torino, Italy; gianlucarosso@hotmail.com; 5 Department of Neurosciences, University of Turin, 10126 Torino, Italy; 6 Department of Psychiatry, Hospital Papa Giovanni XXIII-Bergamo, 24127 Bergamo, Italy; clapal82@gmail.com; 7 Department of Mental Health, Psychiatric Service of Diagnosis and Treatment, Hospital “G. Mazzini”, National Health Service (NHS), ASL 4 Teramo, 64100 Teramo, Italy; domenico.deberardis@aslteramo.it; 8 Department of Neurosciences and Imaging, Chair of Psychiatry, University “G. D’Annunzio”, 66100 Chieti, Italy

**Keywords:** agitation, bipolar disorder, loxapine, psychomotor agitation, schizophrenia

## Abstract

The early and correct assessment of psychomotor agitation (PMA) is essential to ensure prompt intervention by healthcare professionals to improve the patient’s condition, protect healthcare staff, and facilitate future management. Proper training for recognizing and managing agitation in all care settings is desirable to improve patient outcomes. The best approach is one that is ethical, non-invasive, and respectful of the patient’s dignity. When deemed necessary, pharmacological interventions must be administered rapidly and avoid producing an excessive state of sedation, except in cases of severe and imminent danger to the patient or others. The purpose of this brief review is to raise awareness about best practices for the management of PMA in emergency care situations and consider the role of new pharmacological interventions in patients with agitation associated with bipolar disorder or schizophrenia.

## 1. Introduction

Psychomotor agitation (PMA) is characterized by increased psychomotor activity, motor restlessness, and irritability. Individuals with PMA exhibit heightened responsiveness to internal and external stimuli and experience mental tension or altered cognitive function. PMA can occur as a manifestation of a psychiatric disorder (e.g., schizophrenia and bipolar disorder (BD)), central nervous system disease (e.g., Parkinson’s disease, Alzheimer’s disease or dementia), or substance abuse [1,2]. In the context of BD or schizophrenia, patients experiencing an episode of PMA report feeling uneasy, restless, or nervous and have little success controlling or coping with their agitation [3]. PMA symptom progression usually follows a fluctuating course and can include aggression; however, in all cases, escalation can result in unpredictable, dangerous, and violent behavior that requires a rapid response on healthcare workers and clinicians [4]. Accordingly, prompt recognition and immediate management of PMA symptoms are imperative for decreasing the risk of harm to the patient, healthcare staff, and other individuals in the patient’s proximity [5]. It is further essential that clinicians can quickly determine the severity of agitation and differentiate between medical and nonmedical causes to ensure appropriate treatment. The inability to recognize and effectively address PMA behavior can result in the need for coercive measures such as involuntary medication, physical restraint, and seclusion [6,7], all of which are potentially disturbing to the patient. Although international expert recommendations are available for PMA, standardized protocols and clinical support tools for managing PMA episodes in association with a primary psychiatric condition are often not readily available to physicians and other healthcare professionals [1,6]. Here, we provide a brief overview of the management of PMA in emergency care settings (e.g., in an emergency room or inpatient psychiatric context) and offer input regarding the utility of newer pharmacological interventions for patients with agitation associated with BD or schizophrenia.

## 2. Epidemiology

Data regarding the epidemiology of PMA are poorly generalizable and mainly derive from studies conducted in specific care settings or patient populations. The reported prevalence of agitation in psychiatric emergency services is between 4–10% [5,8,9]. Moreover, up to 50% of psychiatric emergency service visits involve patients affected by schizophrenia, BD, or dementia, who are patients in which PMA is considered a common symptom in emergency contexts [10,11]. A study in Spain suggested that about 25% of patients with schizophrenia and 15% of those with BD. Experience at least 1 episode of PMA per year, with a median of 2 episodes per year [12]. In the cross-sectional multicenter STAGE study [13], a reported 4.6% of 7295 psychiatric emergencies were agitation episodes. Sixty-three percent of patients who experienced an episode were male and episodes most commonly occurred in patients with schizophrenia and BD, respectively. Substance abuse was another common diagnosis in patients with agitation episodes but rarely presented as a single disorder and was typically associated with a psychiatric diagnosis [13]. A recent Spanish study analyzing over 355,000 hospital discharge records reported PMA in 1.5% of patients [14]; in this cohort, 52.8% of patients with PMA were male and 78% had two or more comorbidities (versus 45.2–60.1% in the control group). These findings suggest that PMA has a mild predilection towards male sex and is more common in patients with higher disease burden, i.e., multiple comorbidities.

## 3. Definitions and Causes

The Diagnostic and Statistical Manual of Mental Disorders 5 (DSM-5) defines agitation as an excessive motor activity associated with a feeling of inner tension. Motor activity is usually non-productive and repetitious and can include behaviors such as pacing, fidgeting, hand wringing, pulling one’s clothes, and an inability to sit still. Even if aggression and violence are not core agitation features, a progression in agitation severity can lead to aggressive and violent behaviors [15,16]. At the first International Experts’ Meeting on Agitation, agitation was defined as “a state where patients cannot remain still or calm, characterized by internal features such as hyperresponsiveness, racing thoughts, and emotional tension; and external ones, mainly motor and verbal hyperactivity, and communication impairment” [17]. This definition, although not all-inclusive, was developed for its practical utility in a clinical setting. Despite these attempts to define agitation, there is still a lack of agreement regarding an exact definition for this broad and multifactorial syndrome.

One reason explaining the difficulty mentioned above in defining PMA is that many consider it a transnosological syndrome that involves multiple and varied pathological processes and occurs due to a diversity of psychiatric and neurological conditions [18]. Considering the nature of PMA, it may be logical to take a multi-dimensional (and multi-disciplinary) approach to its definition that relates to its psychopathology, going beyond the DSM-5 classification. Agitation is a multifactorial syndrome [1] associated with many illnesses such as psychiatric disorders, intoxication/withdrawal, and other general medical conditions [19].

### Psychiatric Disorders and Comorbid Substance Abuse

The differential diagnosis of agitation accompanying schizophrenia or BD requires a careful evaluation of the patient and consideration of all possible factors contributing to an agitated state. Asking the patient to report on their own state with the administration of psychiatric interviews or self-rating scales can exacerbate agitated behaviors and risk escalation from agitation to aggression or violence [20]. In agitation related to schizophrenia, the patient may experience delusions (e.g., persecutory delusions) and command hallucinations to harm others [21,22]. In BD, agitation often manifests during manic episodes as an excess of the activity or depressive episodes as irritability and fluctuating energy levels [23]. Involuntary motor activity can be a symptom of underlying or comorbid somatic illness or a side effect of prescribed therapies for a patient’s disorder. Akathisia related to PMA can be difficult to distinguish in patients who often report vague, non-specific complaints such as nervousness, inner tension, discomfort, restlessness, itching, and inability to relax. Providers must differentiate between drug-induced akathisia and agitation to avoid increasing antipsychotic or antidepressant treatment doses as an incorrect treatment strategy [24].

PMA frequently emerges in psychiatric patients who engage in recreational substance abuse or develop comorbid substance abuse disorder; this phenomenon is especially well-documented in schizophrenia and BD. An estimated 50% of patients with schizophrenia have a comorbid substance abuse disorder [23,25]. Moreover, patients with BD. who have psychotic symptoms and engage in substance abuse have a higher risk of violent aggression, especially during the manic phase [26]. Substances used can include methamphetamines, opioids, nicotine, marijuana, caffeine, hallucinogenic mushrooms, and cocaine. These substances are frequently used for self-medication (rather than pharmacological therapy). PMA episodes are most likely to emerge due to intoxication or abstinence (e.g., opioid withdrawal) from psychoactive substances. Identifying substance abuse as a cause or exacerbating factor for PMA is critical for guiding subsequent treatment and specifying strategies for long-term management.

Alcohol abuse is another frequent cause of PMA, especially in idiosyncratic reactions, acute intoxication, or withdrawal. Subjects who exhibit PMA associated with alcohol abuse are frequently dependent on the substance and have heavily used alcohol in the long term. PMA can also manifest as the result of mixing alcohol with other substances, resulting in a loss of impulse control and aggressive behaviors.

## 4. PMA Recognition and Assessment

PMA is an evolving phenomenon for which rapid intervention is essential to avoid escalation to violent behavior. Tools for its evaluation must facilitate quick and easy assessment based only on clinical observation without interviewing the patient. These tools should provide scores and cut-offs to assess PMA severity and inform therapeutic decision-making.

The early signs of PMA include changes in different domains [6]. In the presence of one or more of these signs, immediate identification of PMA severity and intervention is necessary to avoid escalation to higher levels:

- Behavioral: inappropriate verbal or motor activity, irritability, impaired self-control, reduced cooperation, aimless wandering, complaining.
- Cognitive: temporal/spatial disorientation, decreased attention, delusion/hallucination, refusal to communicate.
- Physical: weakness, headache, autonomic signs as tachycardia, dyspnea, sweating, tremor, muscle tension.

The progression of agitation symptoms can be visualized as a continuum or S-shaped curve that ranges from calm negativity to evident hostility [27]. On this curve, agitation increases with time from mild to moderate to severe, where each category defines different functional characteristics of the patient state. A mild level of agitation is clinically characterized by fast responses, constant nervous movement, and flashes of anger. Moderate agitation is characterized by verbal ranting, violent responses, and non-cooperative or paranoid behavior. Severe agitation is characterized by verbal and physical aggression, attention deficit, lack of coherence, and predisposition to conflict. For example, signs can evolve from complaints to repetitive phrases or movements, inappropriate or accelerated movements, speaking loudly, prolonged eye contact, invasion of personal space, tense and angry facial expressions, and finally result in verbal threats and openly aggressive behavior. Moreover, agitation may be one of the main indicators of imminent and impulsive suicidal behavior [28,29].

In real-world situations, the classification of agitation symptoms using the scheme described above depends largely on a clinician’s experience and capacity for judgment. In order to permit the more standardized assessment of PMA, several rating scales have been developed to assess symptom escalation and agitation severity, including context-specific scales. A number of scales are currently available for use in psychiatric settings (Table 1). Generally speaking, the positive and negative symptom scale-excited component (PANSS-EC) [30] and clinical global impression-aggression (CGI-A) [20] represent the most suitable choices in emergency psychiatric settings. However, the effective application of these scales in a real-world setting still depends on the observer’s skills for individuating specific behaviors. It is essential to highlight that quantitative assessment of PMA severity cannot be separated from a qualitative evaluation and global assessment of the patient’s situation.

The PANSS, initially developed in 1987 for use in patients with schizophrenia, is composed of 30 items subdivided into three distinct groups: positive symptoms (7 items), negative symptoms (7 items), and general psychopathology symptoms (15 items) [31]. Accordingly, the PANSS permits a broad evaluation of the patient’s psychological state, including factors that interact with positive and negative symptomology. The PANSS-EC (excited component) is a 5-item subscale (poor impulse control, tension, hostility, cooperation, and excitation) in which each item is assigned a point value of 1–5. A total score of >14 with at least >4 points on one item indicates the presence of clinically significant PMA, and >20 corresponds with severe PMA Valid treatment response is defined as a >40% reduction in score within 2 h of treatment. Accordingly, the PANSS-EC permits the standardized evaluation of PMA and effective monitoring of its treatment. The PANNS-EC has been specifically validated for the evaluation of acute psychosis and agitation in emergency psychiatric situations [30].

The CGI is frequently used in clinical research and provides a broad evaluation of global psychological status divided into three parts: severity (CGI-S; scored from 1—not at all ill to 7—among the most extremely ill patients), improvement after treatment (CGI-I; scored from 1—very much improved to 7—very much worse), and level of aggression (CGI-A; evaluated on a scale of 1—not at all aggressive to 5—aggressive behavior). Together, these scales provide a comprehensive evaluation of both patient status and response to treatment. The CGI-A has been validated against the PANSS-EC [20] as a more concise alternative under circumstances in which the PANSS-EC cannot be feasibly implemented.

In cases where a patient presenting to the emergency department is subsequently admitted for observation and treatment, longer assessments may be useful for treatment planning purposes and decrease the likelihood of overlooking organic disease signs. To this extent, the brief agitation rating scale (BARS) is a useful and practical scale for the assessment of agitation in a real-world context. It should be noted that the clinical relevance of these scales is sometimes limited due to the urgency of PMA assessment and management and PMA characteristics; in these cases, severity is often reconstructed *ex post*.

## 5. Management Strategies

The management of PMA in emergency care settings largely depends on the severity of patient symptoms and the care context’s nature. In general, non-pharmacological management should progress from less invasive or coercive measures to more invasive or coercive measures [32]. In many circumstances, behavioral control methods and de-escalation strategies such as verbal intervention, offering food or beverages, and letting patients smoke or offering nicotine replacement are helpful to manage agitation and prevent its escalation into aggression or violence [33]. Therefore, the approach to an agitated patient should start with environmental modification and verbal de-escalation in a manner that provides physical comfort and minimizes external stimuli. Effective management is significantly influenced by the coordination of this response among healthcare professionals and the ability to enact environmental modification in a timely fashion [34]. To this end, another critical goal during initial de-escalation is to ensure the safety of the patient and those around them (e.g., remove or secure all objects that can be potentially harmful) while respecting the patient’s dignity and ensuring that the patient does not feel threatened. Recently, the Project BETA de-escalation workgroup proposed ten domains of verbal de-escalation techniques to help clinicians manage agitated patients [35]. This approach aims to reduce the use of coercive maneuvers and instead make the patient a collaborative subject in his own care project.

Environmental issues and professional attitudes play a key role in managing an agitated patient in an emergency care situation [36]. Environmental and/or organizational measures that can reduce the risk of violence include the implementation of general safety protocols, allocation of a reserved room, use of safety doors and metal detectors, a fast and effective communication system to advise the staff about the admission of a patient with a history of violent behavior, availability of security staff, easy access to a security exit, removal of objects that may be used as weapons, minimizing external stimuli, and continuous observation by staff to remove disruptive individuals when necessary. Professional attitudes are also important for managing agitated patient. A calm, positive, empathetic, and respectful approach by specially trained staff can limit or alleviate agitation [37,38].

In some cases, de-escalation is unsuccessful, and temporarily, physical restraint (PR) may become necessary. The Mental Health Unit Use of Force Act issued in 2018 defines PR as “the use of physical interaction which is proposed to avoid, restrict, or subdue the normal movement of any part of the patient’s body.” However, PR should be used as a last resort and only for limited periods of time as a strategy to prevent negative physical and psychological consequences [39].

Pharmacological intervention becomes necessary when verbal and behavioral methods fail to calm the patient. Before starting any medication, physicians should always attempt to diagnose the most likely cause for agitation, guiding or influencing treatment choice. This is because agitation associated with psychosis, mania, and substance abuse is mainly correlated with alterations in catecholamines and specifically elevated dopaminergic neurotransmission (and should, therefore, be amenable to treatment with anti-dopaminergic medications), whereby decreased gamma-aminobutyric acid (GABA) transmission is the primary characteristic of agitation associated with dementia, depression, and anxiety; it should be treated as such [40].

The ideal treatment for the acute management of PMA in an emergency care setting should: (1) be easy to administer and non-traumatic; (2) quickly calm the patient without excessive sedation; and (3) if pharmacological, have a rapid onset of effect with low pharmacokinetic variability, as well as a low risk of adverse events and drug interactions [1,16,27]. While parenteral administration routes (intramuscular (IM), intravenous) ensure rapid action, they are invasive and considered to be potentially traumatic to a distressed patient. Accordingly, oral administration is the preferred route of medication for pharmacological PMA treatment and parenteral routes are reserved for extreme cases of necessity (e.g., severe agitation). Unfortunately, no single therapeutic option currently available meets the definition described above, resulting in an important unmet clinical need.

## 6. Pharmacological Management

Pharmacological strategies for PMA in emergency care settings should control agitation or violent behavior without producing excessive sedation and should preferably avoid invasive administration routes [41]. The pharmacological management of agitation has traditionally employed three classes of medications: first-generation antipsychotics (FGAs), benzodiazepines (BZDs), and second-generation antipsychotics (SGAs) [33]. Available pharmacological tools and emerging treatment options for PMA are outlined below and summarized in Table 2. In the present review we focused on available parenteral and inhaled medications.

### 6.1. First-Generation Antipsychotics

First-generation antipsychotics (FGAs) such as haloperidol and chlorpromazine have a long history of use for the treatment of agitation in BD. and schizophrenia. The exact mechanism of action for calming agitation is unknown but is attributed in part to a negative effect on dopaminergic transmission in the brain, especially at the D2 receptor, which may act to decrease motor agitation. These agents also typically exert noradrenergic, cholinergic, and histaminergic inhibitory actions. A key benefit of haloperidol is that it is useful for the rapid tranquilization of patients exhibiting aggression associated with psychosis, although the use of haloperidol alone when alternatives are available may be unethical [42]. A recent meta-analysis identified haloperidol plus promethazine or droperidol as safe and effective options for rapid tranquilization [43]. Unfortunately, FGAs can also be associated with unpleasant extrapyramidal side effects (e.g., tremor, slurred speech, akathisia, dystonia), some of which appear after long-term exposure (e.g., tardive dyskinesia). Moreover, some FGAs are structurally similar to GABA and interact with human GABA receptors at high doses [44,45]. Among FGAs used for PMA, haloperidol is the gold standard comparator for most trials, whereas zuclopenthixol is the treatment of choice when long-term treatment is necessary [1].

Loxapine is an FGA approved for the treatment of acute agitation in BD. and schizophrenia. Loxapine blocks D2, D1, and D4 dopamine receptors and also blocks serotonin 5HT2 with the same potency [46]. In 2012, the FDA approved an inhaled formulation of loxapine that uses a specific device (Staccato^®^ One Breath Technology™). This technology quickly vaporizes an excipient-free drug formulation to yield an aerosol that is ideal for inhalation and produces peak plasma levels of loxapine within 2 min. Moreover, administration via inhalation of the Staccato^®^ loxapine aerosol is associated with a lower risk of adverse effects (extrapyramidal symptoms, sedative effects) than other systemically administered antipsychotics [47,48]. The pharmacokinetic profile of inhaled loxapine is similar to that of the intravenous formulation [49,50,51]. In a clinical study of patients with schizophrenia or BD, inhaled loxapine (5 mg or 10 mg) exhibited efficacy for mild-to-moderate agitation with a rapid onset of action as determined using PANSS-EC scores [47]. In the PLACID study [52], inhaled loxapine attenuated agitation was measured with the CGI scale faster than IM aripiprazole in patients with schizophrenia or BD in both hospital and emergency settings. Moreover, patients treated with inhaled loxapine were more often “very” or “extremely” satisfied with treatment than the aripiprazole group. Treatments with high patient satisfaction can promote better physician-patient relationships and stimulate patient empowerment [52].

In a majority of studies reporting safety outcomes in patients with schizophrenia or BD, inhaled loxapine was well tolerated and did not produce excessive sedation [53,54,55]. The most commonly reported adverse effects were mild to moderate and included dysgeusia, throat soreness, and sedation [50,51,52,56]. Severe adverse events were rare and included excessive sedation, akathisia [51], asthenia [52], neck dystonia and oculogyric crisis [50], and dystonia in a patient with a history of jaw clenching secondary to antipsychotic treatment [47]. Inhaled loxapine is not associated with cardiovascular toxicity; however, a primary safety issue is the potential for pulmonary adverse events. Direct inhalation of pharmacological agents into the lungs can increase the risk of developing or worsening asthmatic reactions, wheezing, and bronchospasm [57,58]. Although pulmonary adverse events were uncommon and mild or moderate in reported studies, it is noteworthy that intervention with albuterol was at times necessary to treat wheezing and bronchospasm [50,51,52,56]. Gross et al. [59] demonstrated that inhaled loxapine potentially elicits airway effects such as FEV1 reduction and bronchospasm in subjects with asthma and chronic obstructive pulmonary disease. These effects were usually reversible with a β-agonist bronchodilator such as salbutamol or albuterol. It is reasonable to recommend a brief pulmonary assessment (e.g., medical history and physical examination) before treatment with inhaled loxapine [60]. A short-acting β-agonist bronchodilator should always be readily available in psychiatric emergency settings when choosing this drug to treat PMA [47].

### 6.2. Second-Generation Antipsychotics

SGAs exert a combined inhibitory effect on both dopaminergic (D2) and serotonergic (5-HT2A) neurotransmission. A body of research has demonstrated the efficacy of several agents for the treatment of PMA, including aripiprazole, quetiapine, risperidone, and ziprasidone; however, oral olanzapine is generally preferred for its favorable side effect profile (a main concern being orthostasis), especially in comparison to other IM FGAs such as haloperidol [44,45,61,62]. The new availability of olanzapine as an intranasal formulation and a recent meta-analysis highlighting it as an ideal agent for rapid tranquilization make it particularly attractive for treating episodes of PMA in emergency care settings [43,63]. Risperidone is not necessarily indicated in older patients due to a risk of hypotension [64]. IM aripiprazole is approved for the treatment of agitation associated with schizophrenia or BD, and doses of 5–20 mg have shown the same efficacy as 2.5–10 mg haloperidol for the treatment of PMA [61]. In a randomized control trial of droperidol, ziprasidone, and midazolam for acute undifferentiated agitation in emergency care, ziprasidone was less likely to require rescue medication for adequate sedation than midazolam. However, sedative effects were somewhat delayed compared to other agents [65]. Accordingly, expert guideline recommendations indicate the suitability of oral SGAs as first-line therapy for acute agitation in emergency contexts [2], although an analysis of common practice suggests that these agents are underutilized in emergency care [66].

Orally disintegrating formulations of aripiprazole, olanzapine, and risperidone have been developed, but this administration method does not improve time to onset or offer other notable advantages in terms of safety or efficacy [67,68]. Sublingual asenapine is also approved by the US Food and Drug Administration (FDA) to treat schizophrenia and manic/mixed episodes associated with BD.

### 6.3. Benzodiazepines

Benzodiazepines (BDZs) are a class of sedative drugs that have been used extensively for rapid tranquilization [69]. All benzodiazepines share a common action mechanism and produce a similar range of effects, including anxiolytic, hypnotic, muscle relaxant, and anticonvulsant actions. The action mechanism is related to allosteric modulation of GABA-A receptors that increases the receptor’s affinity for its cognate ligand. For this reason, the central nervous system depressant effects of BDZs are more prominent than those associated with antipsychotic agents [69].

BDZ potency is determined by the affinity of drug-receptor binding. For example, lorazepam and clonazepam have high potency, diazepam has moderate potency, and temazepam has low potency. The time to onset depends on drug lipophilicity, with midazolam and lorazepam being the fastest acting and oxazepam being a slower acting BDZ [70].

Given the fast action of lorazepam, standard treatment for acute psychotic agitation typically involves intramuscular injection of lorazepam and the FGA haloperidol. In one study, oral lorazepam plus risperidone was as effective as this standard treatment, suggesting that an oral BDZ combination may provide effective management of PMA episodes [71]. The combination of haloperidol with BDZs also appears to be safe for acute agitation in emergency contexts [72,73]. In contrast, the administration of intramuscular olanzapine isn’t recommended with BDZs due to potential adverse effects [41].

Diazepam is also used to manage P but should be administered intravenously as it precipitates when used IM. Moreover, its long effect duration and prolonged sedation can be bothersome in some cases.

## 7. Limitations

The present work has several limitations. First, we elected to perform a brief overview of available literature and accordingly did not perform a systematic review or meta-analysis as perhaps this topic merits. However, authors participated in electronic database search and selected articles for inclusion based on their own experience and judgment. Second, the authors were all of the same nationality and work in similar clinical environments, which may have introduced a source of bias. Finally, this article’s recommendations are specifically in emergency care and therefore may not be applicable in all clinical settings.

## 8. Conclusions

The prompt recognition and management of PMA in emergency care settings and clinical settings remain important healthcare providers’ challenges. Differential diagnosis of the underlying cause of the PMA episode and objective grading of its severity are necessary to inform appropriate management. Initial responses include de-escalation and behavioral strategies to calm the patient and make them a cooperative participant in their own care. Invasive treatment modalities (e.g., intramuscular medications) should only be reserved for severe cases of agitation, and when pharmacological intervention is needed, oral or other non-invasive modalities of administration are preferable. Among traditional approaches to the pharmacological management of PMA in emergency care settings, inhaled loxapine is emerging as a beneficial therapy associated with good efficacy, safety, and patient satisfaction.

## Figures and Tables

**Table 1:** Ratings scales for psychomotor agitation in emergency care settings.

Scale	No. Items	Scoring	Total Score Range	Time to Complete
The Agitated Behaviors Mapping Instrument (ABMI)	29 items	1–4	29–203	From 20 min to 1 h observation
The Agitation Severity Scale (ASS)	21 items	0–3	0–63	10 min
The Aggressive Behavior Scale	4 items	0–3	0–12	7 days of observation
The Agitated Behavior Scale	14 items	1–4	14–50	30 min
The Brief Agitation Measure (BAM)	3 items	0–7 (for the past week)	3–21	Few minutes
The Brief Agitation Rating Scale (BARS)	10 items	0–3	0–30	4 days observation
The Broset Violence Checklist (BVC)	6 items	0–1 absent/present	0–6	Few minutes
The Clinical Global Impression Scale for Aggression (CGI-A)	1 item	1–5	1–5	1–2 min
The Cohen-Mansfield Agitation Inventory (CMAI)	4 domains 29 items	1–7 (for the past week)	29–203	20 min
The Historical, Clinical, and Risk Management-20 Violence Risk Assessment Scheme (HCR-20)	3 domains 20 items	N: No/ P: Possibly/ Y: Yes H: in the last 1–6 months C: current episode R: future risk for 1–6 months	Not applicable	From 30 min to a few hours
The Neurobehavioral Rating Scale- Revised (NRS-R)	5 domains 29 items	0–3	0–87	From 20 min to 1 h
The Overt Aggression Scale (OAS)	4 domains 16 items	0–4	0–16	A few minutes
The Overt Agitation Severity Scale (OASS)	3 domains 16 items	1–4	0–120	15 min observation
The Positive and Negative Syndrome Scale-Excited Component (PANSS- EC)	5 items	1–7	5–35	Few minutes
The McNiel-Binder Violence Screening Checklist (VSC)	5 items	0–1 absent/present	0–5	Few minutes
The Pittsburgh Agitation Scale (PAS)	4 items	0–4	0–16	A few minutes
The Ryden Aggression Scale	26 items	0–5	0–125	20 min
The State-Trait Anger Expression Inventory (STAXI)	44 items	0–4	0–132	5–10 min
The Staff Observation Scale (SOAS)	4 events	3–10	24–30	10–15 min of event reporting

**Table 2:** Parenteral and inhaled pharmacological treatments for psychomotor agitation.

Class	Drug	Mode of Admin	Dose Range (mg)	Adverse Effects	Contraindications	Treatment Associations and Recommendations
**FGA**	Haloperidol	IM	5–30	NMS Extrapyramidal side effects Torsade de pointes QT prolongation	Severe cardiovascular disorders History of seizures EEG abnormalities Dementia-related psychosis Parkinson’s disease Haloperidol hypersensitivity	Lorazepam, promethazine, or diphenhydramine (low risk of NMS) FGAs should only be administered during pregnancy if the benefit clearly outweighs the potential risk to the fetus Use with caution in patients < 17 years of age.
		*IV*	*5–20*	*Falls* *Torsade de pointes* *Cardiac arrest* *Sudden death*		
	Chlorpromazine	IM	50–150	Hypotension Falls Pain at the site of injection NMS Extrapyramidal side effects Alpha-adrenergic effects	History of seizures Dementia-related Psychosis	
		*IV*	*25*–*50*	*Prolonged unconsciousness* *Sudden death (for high doses)*		
	Loxapine	Inhalation	9.1–18.2	Extrapyramidal side effects	Asthma Chronic obstructive pulmonary disease	Evidence of use in minors unavailable
	*Zuclopenthixol Acetate*	*IM*	*50*–*150*	*Fatal cardiac events* *Sudden death*	Patients requiring immediate effect onset (delayed onset of about 8 h) Children and adolescents	
	Promazine	IM	50–300	Hypotension Somnolence Dizziness Paralytic ileus Ketoacidosis NMS	Coma Bone marrow depression Pheochromocytoma Central nervous system depression Promazine hypersensitivity	In children ≥ 12 years and adolescents, dosage should not exceed 0.25–0.50 mg/kg
**SGA**	Aripiprazole	IM	10–30	Low risk of extrapyramidal effects Cardiovascular effects	Cardiovascular disorders	Lorazepam The safety and efficacy of aripiprazole injection have not been established in subjects ≤ 17 years
	Ziprasidone	IM	10–40	DRESS	Cardiovascular disorders	The safety and efficacy of ziprasidone injection have not been established in subjects ≤ 17 years
	Olanzapine	IM	10–20	Hypotension Bradycardia Cardiorespiratory depression	Substance or alcohol abuse Contraindicated in association with benzodiazepines	Administration with BDZ. isn’t recommended due to safety considerations. The safety and efficacy of olanzapine injection have not been established in subjects ≤ 17 years
**BZD**	Lorazepam	IM IV	2–8	Respiratory depression Ataxia Excessive sedation Memory impairment Paradoxical disinhibition	Intra-arterial administration Use in neonates or infants Acute narrow-angle glaucoma Severe respiratory insufficiency Alcohol dependence and abuse Sleep apnea	Oral risperidone Use lower dosages in children and adolescents Drug of choice for psychomotor agitation in epilepsy
	Diazepam	IV	10–40	Respiratory depression Ataxia Excessive sedation Memory impairment Paradoxical disinhibition	Intra-arterial administration Use in neonates or infants Acute narrow-angle glaucoma Severe respiratory insufficiency Alcohol dependence and abuse Sleep apnea	Use lower dosages in children and adolescents Useful for psychomotor agitation in epilepsy
Others (when antipsychotics or BDZs are contraindicated)	Sodium valproate	IV	400–1200	Increased liver enzymes Hepatotoxicity Excessive sedation Ataxia	Intra-arterial administration Use in neonates or infants Hepatic disorders Porphyria Coagulopathies Pregnancy and breastfeeding Mitochondrial disorders such as Alpers-Huttenlocher syndrome	IV sodium valproate doesn’t have direct psychiatric indications in the product label Useful for psychomotor agitation in epilepsy

Italicized drugs or modes of administration are not recommended. BZD, benzodiazepine; DRESS, drug reaction with eosinophilia and systemic symptoms; EEG, electroencephalogram; FGA, first-generation antipsychotic; IM, intramuscular; IV, intravenous; NMS, neuroleptic malignant syndrome; SGA, second-generation antipsychotic.
